# Factors Affecting a Short-Term Response to Anti-VEGF Therapy in Diabetic Macular Edema

**DOI:** 10.3390/life11020083

**Published:** 2021-01-25

**Authors:** Ayumi Usui-Ouchi, Asaka Tamaki, Yoshihito Sakanishi, Kazunori Tamaki, Keitaro Mashimo, Toshiro Sakuma, Nobuyuki Ebihara

**Affiliations:** Department of Ophthalmology, Juntendo University Urayasu Hospital, 2-1-1 Tomioka, Urayasu, Chiba 279-0021, Japan; akawata@juntendo.ac.jp (A.T.); ysakani@juntendo.ac.jp (Y.S.); kztamaki@juntendo.ac.jp (K.T.); kmashimo@juntendo.ac.jp (K.M.); t-saku@juntendo-urayasu.jp (T.S.); ebihara@juntendo.ac.jp (N.E.)

**Keywords:** diabetic macular edema, ranibizumab, aflibercept, anatomical response, optical coherence tomography, fluorescein angiography, systemic factors

## Abstract

Diabetic macular edema (DME) is a common cause of visual impairment in patients with diabetes. Although intravitreal anti-vascular endothelial growth factor (VEGF) injections were efficacious in clinical trials, several patients exhibited a poor response. This study aimed to compare clinical features between patients who were susceptible to intravitreal anti-VEGF injections for DME and those who were not. A single-center, retrospective study of 102 such patients was conducted (123 eyes; mean ± standard deviation age, 63.4 ± 10.8 years; 57.8% males). Systemic and ocular data, assessed at baseline and after a month, were compared between good (>20% decrease in central macular thickness (CMT)) and poor (≤20% decrease in CMT) responders using the Mann–Whitney U test/Fisher’s exact test. Eighty-one eyes (65.9%) were good responders. The glycosylated hemoglobin level was higher (*p* = 0.011) in poor (7.5% ± 0.94%) than in good (7.04% ± 1.19%) responders. The foveal avascular zone was larger (*p* = 0.0003) in poor (0.67 ± 0.33 μm^2^) than in good (0.47 ± 0.23 μm^2^) responders. The number of microaneurysms in the pericapillary network was higher (*p* = 0.0007) in poor (2.7 ± 2.2) than in good (1.4 ± 2.0) responders. Baseline glycemic control and macular ischemia may be associated with the short-term response to intravitreal anti-VEGF injections.

## 1. Introduction

Diabetic macular edema (DME) is a common cause of visual impairment in patients with diabetic retinopathy. This microvascular complication is estimated to affect one in 15 patients with diabetes; thus, there are more than 20 million cases worldwide [1,2]. Vascular endothelial growth factors (VEGFs) are known to play an important role in increasing vascular permeability in patients with diabetic retinopathy [3]. Intravitreal anti-VEGF injections are recognized to improve visual outcomes and decrease macular fluid in patients with DME [4,5]. Such agents are the current gold standard in the treatment of DME, and their safety and efficacy have been proven in large randomized clinical trials, as well as in real-world studies [6]. However, the pathogenesis of DME is complex, with multiple factors contributing to its pathophysiology, including angiogenic, inflammatory, hypoxic, and hemodynamic processes that lead to the breakdown of the blood-retinal barrier and leakage of the intraretinal fluid [7]. This may be why certain patients respond moderately or even poorly to anti-VEGF therapy. For example, in two landmark clinical trials, 14.4% (RIDE) and 15.2% (RISE) of patients experienced no improvement or decreased visual acuity at the primary endpoint, even though patients with DME in clinical trials receive far more injections than patients in clinical practice [5,8].

A number of studies have been conducted to examine which factors influence the clinical outcomes of DME treatment with anti-VEGF agents [9,10,11,12,13,14,15,16]. However, the results were inconsistent and most focused on systemic factors, visual acuity, or macular anatomical factors using optical coherence tomography (OCT), but not fluorescein angiography (FA) [9,10,11,12,13,14]. The purpose of this study was to elucidate which clinical features affect the anatomical response to intravitreal anti-VEGF therapy in patients with DME; we discovered that FA findings were highly associated with that response.

## 2. Materials and Methods

### 2.1. Patients

The medical records of consecutive patients who received their first intravitreal anti-VEGF injections for center-involving DME at Juntendo University Urayasu Hospital from March 2014 to October 2015 were evaluated retrospectively. All patients underwent systemic and ophthalmological examination before their first injection. Systemic examination included measurements of blood pressure, serum glycosylated hemoglobin (HbA1c), serum creatinine, and urine protein. Ophthalmological examination included best-corrected visual acuity (BCVA) measurement, intraocular pressure measurement, fundus color photography, OCT (Cirrus HD-OCT; Carl Zeiss Meditec AG, Jena, Germany), and FA (TRC-50DX; Topcon Corp., Tokyo, Japan). BCVA was determined using a Landolt C chart and converted from the decimal system to the logarithm of the minimum angle of resolution (logMAR).

The exclusion criteria were as follows: 1. prior vitreoretinal surgery; 2. any other treatment for DME (such as previous anti-VEGF therapy, topical steroid therapy, or focal/grid laser photocoagulation for DME) within 6 months before the first injection; 3. insufficient quality of OCT or FA; 4. the presence of any retinal diseases other than diabetic retinopathy (such as macular degeneration or retinal vascular occlusions); 5. substantial vitreomacular traction or the presence of an epiretinal membrane. All the procedures and measurements adhered to the tenets of the Declaration of Helsinki of 1975, revised in 2013, and the study was approved by the ethics committee at the Juntendo University Urayasu Hospital.

### 2.2. Intravitreal Anti-VEGF Injections

All intravitreal anti-VEGF injections were performed in the operating room after obtaining patients’ written informed consent. The medications used were either aflibercept (2 mg/0.05 mL) or ranibizumab (0.5 mg/0.05 mL). A single introductory intravitreal injection of aflibercept or ranibizumab was administered, followed by pro re nata injections. OCT was performed one month after injection. The response to anti-VEGF injections was defined as the reduction in central macular thickness (CMT) at one month after injection compared to the CMT before injection. Cases in which CMT reduction was more than 20% were designated to the “good response” group and the others were designated to the “poor response” group.

### 2.3. Imaging

**Figure 1:**
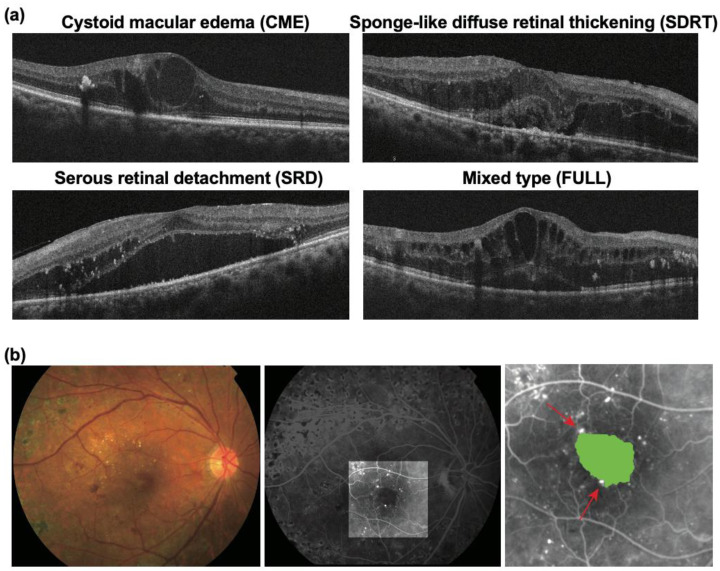
Diabetic macular edema (DME) morphology. (**a**) Representative optical coherence tomography images of four types of DME morphology; (**b**) representative fundus photography (left) and fluorescein angiography images of DME (middle and right). The area colored in green indicates the foveal avascular zone and the red arrows indicate microaneurysms in the pericapillary network.

Cases were classified into four groups, using OCT, based on DME morphology, according to previous reports [17,18]: cystoid macular edema (CME), sponge-like diffuse retinal thickening (SDRT), serous retinal detachment (SRD), and all three factors combined (FULL) (Figure 1a). The foveal avascular zone (FAZ) was determined from the FA images by identifying the innermost capillaries around the fovea (the perifoveal capillary network (PCN)); calculations were performed using Image J, as detailed in a previous report [19] (Figure 1b). Microaneurysms (MAs) in the PCN were counted using both early- and late-phase FA images as previously reported [19] (Figure 1b).

### 2.4. Statistical Analysis

Data are expressed as means ± standard deviation and were analyzed using the Mann–Whitney U-test or Fisher’s exact test. The intensity of correlation between FAZ size and number of MAs in PCN was evaluated by Pearson’s correlation coefficient r. FAZ size and number of MAs in PCN between four groups of DME morphology were evaluated using one-way ANOVA with Tukey’s post hoc test. A *p*-value less than 0.05 was considered statistically significant. All statistical analyses were performed using Prism 6 (GraphPad Software, Inc., San Diego, CA, USA).

## 3. Results

### 3.1. Baseline Characteristics

In total, 102 patients (123 eyes) were included in this study. The average age of the patients was 63.4 ± 10.8 years (range from 29 to 87). Fifty-nine (57.8%) were male and 43 (42.2%) were female. All patients were diagnosed with type 2 diabetes. Baseline characteristics, according to response to anti-VEGF therapy, are summarized in Table 1. The level of HbA1c was significantly lower in good than in poor responders (7.04% ± 1.19% vs. 7.50% ± 0.94%; *p* = 0.011).

### 3.2. Baseline Ocular Characteristics

**Figure 2:**
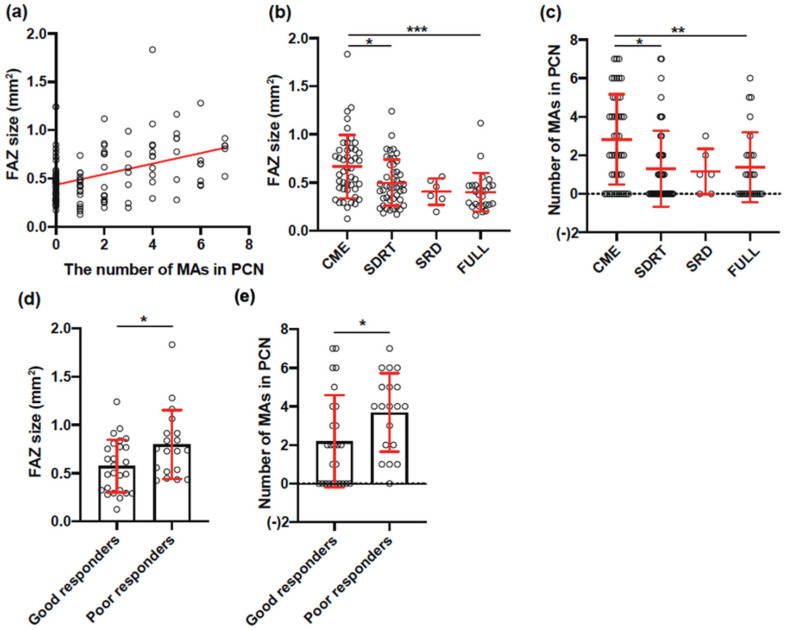
(**a**) The correlation between foveal avascular zone (FAZ) size and the number of microaneurysms (MAs) in the pericapillary network (PCN) (r = 0.42, *p* < 0.0001); (**b**) the FAZ size in each diabetic macular edema type; (**c**) the number of MAs in the PCN in each diabetic macular edema type; (**d**) the FAZ size among CME eyes that were good and poor responders; (**e**) the number of MAs in the PCN among CME eyes that were good and poor responders. * *p* < 0.05, ** *p* < 0.01, *** *p* < 0.001. The error bars indicate standard deviation. CME, cystoid macular edema; SDRT, sponge-like diffuse retinal thickening; SRD, serous retinal detachment; FULL, combination of CME, SDRT, and SRD.

We divided patients into four groups based on DME morphology using OCT according to previous reports [17,18]; a representative OCT image for each group is presented in Figure 1a. FAZ size and the number of MAs were determined from the FA images according to a previous report [19]; representative FA images are presented in Figure 1b. Baseline ocular characteristics according to response to anti-VEGF therapy are summarized in Table 2. The FAZ in poor responders was significantly larger than that in good responders (0.67 ± 0.33 μm^2^ vs. 0.47 ± 0.23; *p* = 0.0003). The number of MAs in the PCN in poor responders was also significantly higher than that in good responders (2.7 ± 2.2 vs. 1.4 ± 2.0; *p* = 0.0007). There was a significant correlation between the number of MAs in the PCN and FAZ size (r = 0.42, *p* < 0.0001) (Figure 2a). Among poor responders, CME was the most common type of macular edema (47.6%) (Table 3). On the other hand, SDRT was the most common type among good responders (39.5%) (*p* = 0.060). Eyes with CME had a significantly higher number of MAs in the PCN (*p* = 0.0024) and a significantly larger FAZ size (*p* = 0.0003) than eyes with other types of macular edema (Figure 2b,c). Moreover, among eyes with CME, poor responders had significantly larger FAZs and a significantly higher number of MAs in the PCN than good responders (*p* = 0.039 and *p* = 0.024) (Figure 2d,e).

### 3.3. Response to Initial Anti-VEGF Therapy

In total, 34.1% (42) of eyes were poor responders, with a mean reduction in CMT of 6.13 ± 8.93%, and 65.9% (81) were good responders, with a mean reduction in CMT of 43.00 ± 14.55%. Altogether, 68 eyes (55.3%) underwent intravitreal ranibizumab injection and 55 (44.7%) underwent intravitreal aflibercept injection (Table 3). Of eyes treated with ranibizumab and aflibercept, 66.2% and 65.5%, respectively, were good responders (*p* > 0.999) (Table 3). LogMAR visual acuity at one month after anti-VEGF therapy was 0.38 ± 0.25 in good and 0.42 ± 0.34 in poor responders.

## 4. Discussion

We investigated the factors affecting the anatomical outcome in patients with DME treated with ranibizumab or aflibercept. Similar studies have been performed previously [9,10,11,12,13,14,15,16]; however, to our knowledge, no detailed examination of FAZ size and the number of MAs, using FA images, has been reported for such a study.

In this study, we discovered that a high baseline HbA1c level, a large baseline FAZ, and a high baseline number of MAs in the PCN were associated with a poor response to anti-VEGF injections. The influence of baseline HbA1c on the outcome of anti-VEGF therapy for patients with DME was controversial in previous studies [10,11,16,20,21,22]. Chen et al. [16] discovered that HbA1c was a prognostic factor for visual outcome only in eyes that responded to intravitreal ranibizumab injection. In addition, Matsuda et al. [11] observed a statistically significant improvement in visual acuity in patients with HbA1c ≤7.0% after anti-VEGF therapy, whereas a statistically significant but smaller improvement in visual acuity was observed in patients with HbA1c >7.0%. On the other hand, Singh et al. [20] revealed that vision improvement upon ranibizumab injection was not affected by systemic factors such as HbA1c, renal function, or blood pressure. A post-hoc analysis of the RIDE/RISE trials demonstrated that improvement in visual acuity, remission of macular edema, and improvement in the severity of diabetic retinopathy following ranibizumab treatment appeared to be independent of baseline HbA1c levels [10]. In our study, the HbA1c level, but not the presence of diabetic nephropathy and hypertension, was associated with the response to anti-VEGF therapy. Patients with a lower HbA1c level tended to be more susceptible to anti-VEGF therapy than those with a higher HbA1c level were, suggesting that blood-sugar control may play a role in the anatomical outcome of anti-VEGF treatment for patients with DME.

In previous reports [23,24], macular ischemia (a FAZ larger than 1000 μm^2^) was statistically significantly associated with a worse visual, but not anatomical, outcome. However, in that study, those without macular ischemia were categorized into one group. In our study, most of the subjects (117/123, 95.1%) did not have macular ischemia according to that definition; we considered the whole range of FAZ sizes, without categorization, and revealed that FAZ size was associated with the anatomical response to anti-VEGF therapy. We also demonstrated that the number of MAs was associated with the response to anti-VEGF therapy. Murakami et al. [19] reported that, compared with eyes with SRD or SDRT, those with CME had more MAs in the PCN and a larger FAZ upon FA. In our study, CME was the most common of the four DME types among poor responders (47.6%), while SDRT was the most common type among good responders (39.5%). We have also demonstrated that FAZ size and the number of MAs in the PCN were higher in eyes with CME than those in eyes with SDRT, SRD, or FULL. Moreover, in our study, there was a correlation between the size of the FAZ and the number of MAs in the PCN.

In terms of OCT-based morphologic findings, bevacizumab appears less effective in the SRD type of DME than in the others, although Kim et al. reported that changes in CMT and BCVA from baseline were not significantly different between groups at 12 months [17,25]. Seo et al. [14] divided their patients into only three groups based on DME type: SDRT, CME, and SRD. They discovered that ranibizumab was most effective for treatment of the SDRT type of DME, and the fewest injections were needed for that type. In our study, SDRT was also the most common DME type among good responders. Shimura et al. [17] reported that the effectiveness of bevacizumab in reducing macular edema was greater in the SDRT and CME groups than in the FULL and SRD groups. However, they also mentioned that two subgroups of CME could be distinguished according to their response to anti-VEGF therapy. Taken together, we suggest that eyes with CME are the least susceptible to anti-VEGF therapy, corresponding to their large number of MAs in the PCN and their large FAZs. Indeed, in the eyes with CME, poor responders had a larger FAZ and a higher number of MAs in the PCN than good responders did in our study. However, it is unclear how FAZ size and the number of MAs in the PCN interact with CME. It has been shown in several publications that the histopathology of CME consists of liquefactive necrosis of Müller cells, which may lead to cystoid spaces [19,26,27]. Murakami et al. [19] hypothesized that cystoid spaces, in which neuroglial cells have been necrotized, would not produce the growth factors necessary for the maintenance of the inner blood-retinal barrier. They also hypothesized that the imbalance between intra- and extramural pressure in the capillaries around cystoid spaces would result in weak points in the capillary wall, leading to the development of MAs. From that perspective, we hypothesize that CME characterized by MAs in the PCN is caused by the necrotization of neuroglia, and that this type CME is less susceptible to anti-VEGF therapy, which does not rescue neuroglial necrosis. Further investigation to verify this hypothesis is warranted.

We separated patients into good and poor responders according to the percentage of CMT reduction between baseline and one month after primary intravitreal anti-VEGF injection. Several previous studies have also defined the treatment response according to anatomical outcomes after intravitreal anti-VEGF therapy [12,28,29]. For example, Bressler et al. [12] divided subjects into four categories. Similar to that in our study, they set the CMT reduction threshold to 20%, but they analyzed the reduction at three different time points during the first treatment year. Koyanagi et al. [29] defined “immediate responders” as those with a more than 25% decrease in CMT at three months after treatment, as opposed to “delayed responders,” who did not exhibit such a decrease at the same time point. We defined “good responders” as those with a more than 20% decrease in CMT at one month after initial treatment. Shah et al. [30] revealed that anatomical outcomes after one injection were predictive of OCT findings at three months. This suggests that anatomical outcomes at one month after injection may indicate whether anti-VEGF therapy is effective, allowing for an early switch, if necessary, to e.g., corticosteroid treatment. Indeed, Cho et al. [31] evaluated the short-term efficacy of intravitreal bevacizumab and posterior sub-Tenon triamcinolone injections in eyes with different DME types. They discovered that the reduction of intraretinal edema, such as CME, was greater with triamcinolone than with bevacizumab. As anti-VEGF therapy is expensive, it would be helpful to be able to predict whether a patient will respond thereto in the early phases of treatment.

OCT angiography (OCTA) is a relatively new technology and a useful tool to evaluate microvasculature. Although it is a noninvasive modality and can be used to produce three-dimensional images of the retinal microvasculature, it does not allow the detection of all MAs; MAs are typically easier to visualize using FA [32,33]. On the other hand, in terms of the FAZ size, OCTA may be preferable to FA as FAZ edges are easier to delineate using OCTA than FA, and FAZ size appears larger using FA than OCTA [34]. In this study, we measured the FAZ size and the number of MAs in the PCN using FA rather than OCTA to improve the detection of MAs. Lee et al. [35] investigated the structural integrity of the superficial and deep capillary plexuses using OCTA in patients with DME, as well as their association with the response to anti-VEGF treatment. They observed that poor responders tended to exhibit more MAs in the deep capillary plexus and a larger FAZ size. Our FA results are consistent with the OCTA results of Lee et al. [35] in terms of the number of MAs and the FAZ size, despite using different modalities and despite the fact that we evaluated MAs only in the PCN whereas Lee et al. evaluated MAs using macular images. Moreover, we analyzed the interaction between FA findings and DME morphology associated with the response to anti-VEGF therapy, which distinguishes our study from previous studies. Further analyses using both FA and OCTA are needed.

The limitations of this study are inherent to its retrospective and short-term nature. The small sample size of our study may have attenuated the statistical power for detecting differences between the groups. To determine the efficacy of intravitreal anti-VEGF treatment for patients with DME, prospective studies with larger sample sizes are needed. Additionally, as the response to anti-VEGF treatment has been defined in many ways in different studies, it may be difficult to compare our study to those using different anatomic and visual criteria.

In conclusion, this study revealed that glycemic control and the level of ischemia in the macula at baseline may be associated with the short-term response to the first intravitreal anti-VEGF injection, and may help predict which patients with DME should be considered for a change to anti-inflammatory treatment.

## Figures and Tables

**Table 1:** Comparison of baseline characteristics between good and poor responders.

Parameter	Total (N = 123 Eyes, 102 Patients)	Good Responders (N = 81 Eyes, 64 Patients) ^1^	Poor Responders (N = 42 Eyes, 38 Patients) ^2^	*p*-Value ^3^
Age	63.4 ± 10.8	62.1 ± 11.4	65.9 ± 9.2	0.061
Gender (male:female)	59:43	43:21	21:17	0.290
HbA1c (%)	7.2 ± 1.1	7.0 ± 1.2	7.5 ± 0.9	0.011
Hypertension (yes:no)	77:46	52:29	25:17	0.695
Diastolic blood pressure (mmHg)	135.9 ± 20.6	136.3 ± 22.3	135.1 ± 17.0	0.362
Systolic blood pressure (mmHg)	72.5 ± 12.4	73.2 ± 13.4	71.0 ± 10.1	0.350
Nephropathy (yes:no)	48:75	35:46	13:29	0.243
Insulin therapy (yes:no)	56:67	34:47	22:20	0.340

^1^ Cases in which reduction in central macular thickness was >20%. ^2^ Cases in which reduction in central macular thickness was ≤20%. ^3^ Mann–Whitney U test or Fisher’s exact test. Continuous variables are indicated as means ± standard deviation. HbA1c, serum glycosylated hemoglobin.

**Table 2:** Comparison of baseline ocular characteristics between good and poor responders.

Parameters		Total (N = 123)	Good Responders (N = 81) ^1^	Poor Responders (N = 42) ^2^	*p*-Value ^3^
	LogMAR visual acuity (baseline)	0.54 ± 0.31	0.52 ± 0.27	0.58 ± 0.37	0.416
	PDR:NPDR	28:95	22:59	6:36	0.119
	PRP (yes:no)	95:28	65:16	30:12	0.364
OCT	CME	45 (36.6%)	25 (30.9%)	20 (47.6%)	0.060
	SDRT	46 (37.4%)	32 (39.5%)	14 (33.3%)	
	SRD	6 (4.9%)	4 (4.9%)	2 (4.8%)	
	FULL	26 (21.1%)	20 (24.7%)	6 (14.3%)	
FA	FAZ size (mm^2^)	0.54 ± 0.28	0.47 ± 0.23	0.67 ± 0.33	0.0003
	Number of MAs in the PCN	1.9 ± 2.2	1.4 ± 2.0	2.7 ± 2.2	0.0007

^1^ Cases in which reduction in central macular thickness was >20%. ^2^ Cases in which reduction in central macular thickness was ≤20%. ^3^ Mann–Whitney U test or Fisher’s exact test. Continuous variables are indicated as means ± standard deviation. LogMAR, logarithm of the minimum angle of resolution; PDR, proliferative diabetic retinopathy; NPDR, non-PDR; PRP, panretinal photocoagulation; OCT, optical coherence tomography; FA, fluorescein angiography; CME, cystoid macular edema; SDRT, sponge-like diffuse retinal thickening; SRD, serous retinal detachment; FULL, combination of CME, SDRT, and SRD; FAZ, foveal avascular zone; MA, microaneurysm; PCN, perifoveal capillary network.

**Table 3:** Categorization of subjects according to response to initial anti-VEGF therapy.

Parameter	Good Responders (N = 81) ^1^	Poor Responders (N = 42) ^2^	*p*-Value ^3^
Ranibizumab: aflibercept (%)	66.2:65.5	33.8:34.5	>0.999
Baseline CMT (μm)	567.14 ± 164.31	517.54 ± 105.64	0.156
CMT one month after anti-VEGF therapy (μm)	314.07 ± 104.00	484.07 ± 98.92	<0.0001
% reduction	43.00 ± 14.55	6.13 ± 8.93	-

^1^ Cases in which reduction in central macular thickness was >20%. ^2^ Cases in which reduction in central macular thickness was ≤20%. ^3^ Mann–Whitney U test or Fisher’s exact test. Continuous variables are indicated as means ± standard deviation. VEGF, vascular endothelial growth factor; CMT, central macular thickness.

## Data Availability

The data presented in this study are available on request from the corresponding author. The data are not publicly available due to privacy.

## References

[1] Yau J.W., Rogers S.L., Kawasaki R., Lamoureux E.L., Kowalski J.W., Bek T., Chen S.-J., Dekker J.M., Fletcher A., Grauslund J. (2012). Global Prevalence and Major Risk Factors of Diabetic Retinopathy. Diabetes Care.

[2] Tan G.S., Cheung N., Simó R., Cheung G.C.M., Wong T.Y. (2017). Diabetic macular oedema. Lancet Diabetes Endocrinol..

[3] Adamis A.P., Miller J.W., Bernal M.-T., D’Amico D.J., Folkman J., Yeo T.-K., Yeo K.-T. (1994). Increased Vascular Endothelial Growth Factor Levels in the Vitreous of Eyes With Proliferative Diabetic Retinopathy. Am. J. Ophthalmol..

[4] Heier J.S., Korobelnik J.-F., Brown D.M., Schmidt-Erfurth U., Edoardo M., Midena E., Boyer D., Terasaki H., Kaiser P.K., Marcus D.M. (2016). Intravitreal Aflibercept for Diabetic Macular Edema. Ophthalmology.

[5] Nguyen Q.D., Brown D.M., Marcus D.M., Boyer D., Patel S., Feiner L., Gibson A., Sy J.P., Rundle A.C., Hopkins J.J. (2012). Ranibizumab for Diabetic Macular Edema. Ophthalmology.

[6] Mitchell P., Wong T.Y. (2014). Management Paradigms for Diabetic Macular Edema. Am. J. Ophthalmol..

[7] Das A., McGuire P.G., Rangasamy S. (2015). Diabetic Macular Edema: Pathophysiology and Novel Therapeutic Targets. Ophthalmology.

[8] Dugel P.U., Layton A., Varma R.B. (2016). Diabetic Macular Edema Diagnosis and Treatment in the Real World: An Analysis of Medicare Claims Data (2008 to 2010). Ophthalmic Surg. Lasers Imaging Retin..

[9] Sophie R., Lu N., Campochiaro P.A. (2015). Predictors of Functional and Anatomic Outcomes in Patients with Diabetic Macular Edema Treated with Ranibizumab. Ophthalmology.

[10] Bansal A.S., Khurana R.N., Wieland M.R., Wang P.-W., Van Everen S.A., Tuomi L. (2015). Influence of Glycosylated Hemoglobin on the Efficacy of Ranibizumab for Diabetic Macular Edema. Ophthalmology.

[11] Matsuda S., Tam T., Singh R.P., Kaiser P., Petkovsek D., Carneiro G., Zanella M.T., Ehlers J.P. (2014). The impact of metabolic parameters on clinical response to VEGF inhibitors for diabetic macular edema. J. Diabetes Complicat..

[12] Bressler S.B. (2012). Factors Associated With Changes in Visual Acuity and Central Subfield Thickness at 1 Year after Treatment for Diabetic Macular Edema with Ranibizumab. Arch. Ophthalmol..

[13] Koytak A., Altinisik M., Sari E.S., Artunay O., Akkan J.C.U., Tuncer K. (2013). Effect of a single intravitreal bevacizumab injection on different optical coherence tomographic patterns of diabetic macular oedema. Eye.

[14] Seo K.H., Yu S.-Y., Kim M., Kwak H.W. (2016). Visual and morphologic outcomes of intravitreal ranibizumab for diabetic macular edema based on optical coherence tomography patterns. Retina.

[15] Channa R., Sophie R., A Khwaja A., Do D.V., Hafiz G., Nguyen Q.D., A Campochiaro P., Abraham P., Green B., The READ-2 Study Group (2013). Factors affecting visual outcomes in patients with diabetic macular edema treated with ranibizumab. Eye.

[16] Chen Y.-P., Wu A.-L., Chuang C.-C., Chen S.-N. (2019). Factors influencing clinical outcomes in patients with diabetic macular edema treated with intravitreal ranibizumab: Comparison between responder and non-responder cases. Sci. Rep..

[17] Shimura M., Yasuda K., Yasuda M., Nakazawa T. (2013). Visual outcome after intravitreal bevacizumab depends on the optical coherence tomographic patterns of patients with diffuse diabetic macular edema. Retina.

[18] Otani T., Kishi S., Maruyama Y. (1999). Patterns of diabetic macular edema with optical coherence tomography. Am. J. Ophthalmol..

[19] Murakami T., Nishijima K., Sakamoto A., Ota M., Horii T., Yoshimura N. (2011). Foveal Cystoid Spaces Are Associated with Enlarged Foveal Avascular Zone and Microaneurysms in Diabetic Macular Edema. Ophthalmology.

[20] Singh R.P., Habbu K., Ehlers J.P., Lansang M.C., Hill L., Stoilov I. (2016). The Impact of Systemic Factors on Clinical Response to Ranibizumab for Diabetic Macular Edema. Ophthalmology.

[21] Wykoff C.C., Elman M.J., Regillo C.D., Ding B., Lu N., Stoilov I. (2016). Predictors of Diabetic Macular Edema Treatment Frequency with Ranibizumab During the Open-Label Extension of the RIDE and RISE Trials. Ophthalmology.

[22] Kim T.K., Shin H.Y., Kim S.Y., Lee Y.C., Lee M.Y. (2017). Factors Influencing Intravitreal Bevacizumab and Triamcinolone Treatment in Patients with Diabetic Macular Edema. Eur. J. Ophthalmol..

[23] Chung E.J., Roh M., Kwon O.W., Koh H.J. (2008). Effects of Macular Ischemia on the Outcome of Intravitreal Bevacizumab Therapy for Diabetic Macular Edema. Retina.

[24] Douvali M., Chatziralli I.P., Theodossiadis P.G., Chatzistefanou K.I., Giannakaki E., Rouvas A.A. (2014). Effect of Macular Ischemia on Intravitreal Ranibizumab Treatment for Diabetic Macular Edema. Ophthalmology.

[25] Kim M., Lee P., Kim Y., Yu S.-Y., Kwak H.-W. (2011). Effect of Intravitreal Bevacizumab Based on Optical Coherence Tomography Patterns of Diabetic Macular Edema. Ophthalmology.

[26] Tso M.O. (1982). Pathology of Cystoid Macular Edema. Ophthalmology.

[27] Fine B.S., Brucker A.J. (1981). Macular Edema and Cystoid Macular Edema. Am. J. Ophthalmol..

[28] Rayess N., Rahimy E., Ying G.-S., Bagheri N., Ho A.C., Regillo C.D., Vander J.F., Hsu J. (2015). Baseline Choroidal Thickness as a Predictor for Response to Anti–Vascular Endothelial Growth Factor Therapy in Diabetic Macular Edema. Am. J. Ophthalmol..

[29] Koyanagi Y., Yoshida S., Kobayashi Y., Kubo Y., Nakama T., Ishikawa K., Nakao S., Hisatomi T., Ikeda Y., Oshima Y. (2018). Visual Outcomes Based on Early Response to Anti-Vascular Endothelial Growth Factor Treatment for Diabetic Macular Edema. Ophthalmology.

[30] Shah A.R., Yonekawa Y., Todorich B., Van Laere L., Hussain R., Woodward M.A., Abbey A.M., Wolfe J.D. (2017). Prediction of Anti-VEGF Response in Diabetic Macular Edema After 1 Injection. J. Vitr. Dis..

[31] Cho Y.J., Lee D.H., Kim M. (2018). Optical coherence tomography findings predictive of response to treatment in diabetic macular edema. J. Int. Med. Res..

[32] Salz D.A., De Carlo T.E., Adhi M., Moult E.M., Choi W., Baumal C.R., Witkin A.J., Duker J.S., Fujimoto J.G., Waheed N.K. (2016). Select Features of Diabetic Retinopathy on Swept-Source Optical Coherence Tomographic Angiography Compared With Fluorescein Angiography and Normal Eyes. JAMA Ophthalmol..

[33] Ishibazawa A., Nagaoka T., Takahashi A., Omae T., Tani T., Sogawa K., Yokota H., Yoshida A. (2015). Optical Coherence Tomography Angiography in Diabetic Retinopathy: A Prospective Pilot Study. Am. J. Ophthalmol..

[34] Peres M.B., Kato R.T., Kniggendorf V.F., Cole E.D., Onal S., Torres E., Louzada R., Belfort R., Duker J.S., Novais E.A. (2016). Comparison of Optical Coherence Tomography Angiography and Fluorescein Angiography for the Identification of Retinal Vascular Changes in Eyes With Diabetic Macular Edema. Ophthalmic Surg. Lasers Imaging Retin..

[35] Lee J., Gil Moon B., Cho A.R., Yoon Y.H. (2016). Optical Coherence Tomography Angiography of DME and Its Association with Anti-VEGF Treatment Response. Ophthalmology.

